# MiR-214-3p, a novel possible therapeutic target for the pathogenesis of osteoarthritis

**DOI:** 10.1016/j.ebiom.2021.103300

**Published:** 2021-03-25

**Authors:** Antonella Fioravanti, Sara Tenti, Sara Cheleschi

**Affiliations:** Department of Medicine, Surgery and Neuroscience, Rheumatology Unit, Azienda Ospedaliera Universitaria Senese, Policlinico Le Scotte, 53100 Siena, Italy

Osteoarthritis (OA) is the most common musculoskeletal disease and its prevalence is rising due to the increasing obesity and life span of the general population [1]. The main symptoms of the disease are chronic pain, functional limitation, instability and deformity, leading to a considerable impairment of quality of life; globally, OA represents the major contributor of disability in older people [1]. Given the high prevalence and the attendant disability, OA causes a considerable burden for individual and society [1].

The destruction of articular cartilage constitutes the main feature of the disease; the structural breakdown of extracellular matrix components seems to be caused by a predominance of catabolic activities on anabolic processes of chondrocytes [2]. Unfortunately, the pathophysiology of OA has not fully understood and multiple factors ranging from aging to biomechanical and biochemical stimuli contribute to the development and progression of the disease [3].

To date, there are no available disease-modifying drugs to prevent or to slow OA progression. The discovery of the crucial pathways characterizing OA could offer new opportunity to identify compounds potentially able to reduce or stop the disease progression [4].

MicroRNA (miRNA) are a class of small non-coding ribonucleic acids of 22–25 nucleotides involved in the regulation of 30% of the human genome. They are involved in cell differentiation and homeostasis, and modifications of their expression have been linked to different pathological conditions including cancer, cardiovascular diseases, diabetes mellitus, rheumatic and neurological disorders [5].

Growing evidence demonstrated an altered expression of a number of miRNA in OA and their involvement in the regulation of cartilage homeostasis and mechanotransduction [6,7]. Thus, miRNA can represent promising biomarkers and potential therapeutic targets of the disease.

In a recent paper by Cao et al. [8], published in EBioMedicine, it has been reported the data, obtained in human OA and mice primary chondrocyte cultures and in experimental animal model (adult male C57BL/6 mice), on the expression of miR-214-3p and on the possible implication of nuclear factor (NF)-kB pathway in miR-214-3p-mediated effects.

In particular, the Authors showed a decreased expression of miR-214-3p in chondrocytes incubated with Interleukin-1β (IL-1β), the main cytokine implicated in OA pathogenesis. This result was strengthened by the down-regulated expression of the miR-214-3p found in damaged cartilage tissue in comparison to undamaged regions of articular cartilage obtained from human OA patients. Then, to deeper investigate the role of this miRNA in cartilage degradation, the cells were transiently transfected with miR-214-3p specific inhibitor or mimic in presence or in absence of IL-1β. The results showed that the over-expression of miR-214-3p partially counteracts the negative effects of IL-1β-induced apoptosis and extracellular matrix degradation.

The role of NF-kB signaling in inflammatory and degrading processes of OA is well known, while the regulation of the inhibitor of NF-kB kinase (IKK) and in particular of the catalytic subunit IKKβ is poor understood [9]. In the present study, the Authors showed, for the first time, that miR-214-3p directly targeted IKKβ in chondrocytes. In addition, the decreased expression of miR-214-3p observed in damaged OA cartilage contributed to the up-regulation of IKKβ subunit and, in turn, to the activation of NF-kB pathway.

Finally, the key function of miR-214-3p in OA development and progression was analyzed in non surgical mice using intra-articular injection of miR-214-3p antagomir and in OA mice with intra-articular injection of miR-214-3p agomir. Data from these experiments showed a deleterious effect of miR-214-3p antagomir in cartilage metabolism and in synovial inflammation. On the contrary, intra-articular injection of miR-214-3p agomir attenuated synovitis, decreased apoptosis and catabolic cartilagineous processes.

Collectively, these results demonstrated a decreased expression of miR-214-3p in OA chondrocytes stimulated by IL-1β, while an over-expression of this miRNA counteracted the negative effects of IL-1β on apoptosis and matrix degradation. The data obtained *in vitro* were further corroborated by *in vivo* results on animal model. Lastly, the study by Cao et al. [8] showed that miR-214-3p significantly suppressed the activation of NF-kB pathway by targeting IKKβ. These findings suggest a protective effect of miR-214-3p in cartilage metabolism and degradation, and the possible involvement of this miRNA in OA development and progression. Furthermore, these results provide a theoretical basis to target the miR-214-3p/IKKβ/NF-kB axis for the prevention and treatment of OA and should be paid particular attention considering the recent data about new systemic delivery developed to transport miRNA [10].

In conclusion, the paper by Cao et al. [8], for the first time, demonstrates the implication of miR-214-3p in cartilage degradation via NF-kB pathway and highlights the relevance of this miRNA as potential therapeutic target for OA.

However, additional experiments on *in vitro* and *in vivo* models are recommended to confirm these data, to clarify the mechanism by which the expression of miR-214-3p is reduced in OA chondrocytes, to evaluate its possible usefulness as biomarker of the disease or its therapeutic application.

## Contributors

Literature search: A.F., S.T. and S.C.; Data collection: A.F. and S.C.; Data interpretation: A.F. and S.C; Writing: A.F., S.T. and S.C. The final manuscript has been seen and approved by all the Authors.

## Declaration of Competing Interest

The authors declare no conflict of interest.
